# Development of an m6A subtype classifier to guide precision therapy for patients with bladder cancer

**DOI:** 10.7150/jca.99483

**Published:** 2024-08-13

**Authors:** Ganghua Zhang, Jingxin Yang, Jianing Fang, Rui Yu, Zhijing Yin, Guanjun Chen, Panpan Tai, Dong He, Ke Cao, Jiaode Jiang

**Affiliations:** 1 Department of Oncology, Third Xiangya Hospital, Central South University, Changsha, China.; 2 Staff Hospital of Central South University, Central South University, Changsha, China.; 3 Department of Neurosurgery, Third Xiangya Hospital, Central South University, Changsha, China.

**Keywords:** bladder cancer, m6A, tumor immune microenvironment, artificial neural network classifier, treatment responsiveness, prognosis.

## Abstract

**Purpose:** Bladder cancer (BLCA) is a highly heterogeneous tumor. We aim to construct a classifier from the perspective of N6-methyladenosine methylation (m6A) to identify patients with different prognostic risks and treatment responsiveness for precision therapy.

**Methods:** Data on gene expression profile, mutation, and clinical characteristics were mainly obtained from the TCGA-BLCA cohort. Unsupervised clustering was performed to construct m6A subtypes. The tumor microenvironment (TME) landscapes were explored by using ssGSEA, ESTIMATE, and MCPcounter algorithms. K-M survival curves and Cox regression analysis were used to demonstrate the significance of m6A subtypes in predicting prognosis. pRRophetic, oncoPredict, and TIDE algorithms were used to evaluate responsiveness to antitumor therapy. A classifier of m6a subtypes was finally developed based on random forest and artificial neural network (ANN).

**Results:** The two m6A subtypes have significantly different m6A-related gene expression profiles and mutational landscapes. TME analysis showed a higher level of stromal and Inhibitory immune components in subtype B compared with subtype A. The m6A subtype is a clinically independent prognostic predictor of BLCA, subtype B has a poorer prognosis. Drug sensitivity analysis showed that subtype B has lower IC50 values and AUC values for cisplatin and docetaxel. Efficacy assessment showed significantly poorer radiotherapy efficacy and lower immunotherapy responsiveness in subtype B. We finally constructed an ANN classifier to accurately classify BLCA patients into two m6A subtypes.

**Conclusion:** Our study developed a classifier for identifying subtypes with different m6A characteristics, and BLCA patients with different m6A subtypes have significantly different prognosis and responsiveness to antitumor therapy.

## Introduction

According to the latest oncology statistics, bladder cancer (BLCA) remains the most common urological malignancy worldwide, accounting for approximately 4% of new cancers and 3% of cancer-related deaths 1. Moreover, BLCA poses a serious threat to people's life and health due to its rapid metastasis and easy recurrence, and early detection means a better prognosis. BLCA has different pathological types, molecular subtypes, and multiple pathogenic pathways, and there are many variations in pathogenesis and prognosis 2, 3. In recent years, targeted therapies represented by erdafitinib and enfortumab and immunotherapy represented by anti-PD-1/PD-L1 have changed the treatment landscape of BLCA, and patients have more choices besides surgery, radiotherapy, and platinum-containing chemotherapy. However, both conventional and emerging therapies have similarly demonstrated large response heterogeneity among patients with BLCA 4, which cannot be satisfactorily explained by existing predictors and molecular typing. Therefore, it is essential to find new methods for risk stratification, identifying populations with different prognostic and treatment responsiveness profiles, and ultimately achieving precision medicine.

In eukaryotes, N6-methyladenosine (m6A) is one of the most abundant modifications in mRNAs^5^, which is involved in almost all phases of the RNA cycle and affects oncogene expression through the regulation of transcription, maturation, translation, degradation, and stability of mRNAs^6^, which in turn regulates the expression of oncogenes from various perspectives such as cancer stem/initiating cell pluripotency 7, cell differentiation^8^, cell proliferation, migration, angiogenesis, and tumor microenvironment^9^, ^10^ to regulate cancer progression. Studies have shown that m6A plays an important role in the genesis and progression of BLCA. For example, METTL3, as an m6A writer, on the one hand, can accelerate the maturation of premiR221/222 in an m6A-dependent manner, leading to a reduction of PTEN and ultimately to the proliferation of BLCA 11. On the other hand, it can mediate m6A modification of PD-L1 thereby promoting immune escape in BLCA 12. METTL14 promotes lncDBET expression through m6A modification leading to active lipid metabolism and malignant progression of BLCA cells 13. m6A modification of BLCACAT3 promotes its expression leading to angiogenesis and hematogenous metastasis of BLCA 14. In addition, an increasing number of studies have begun to explore the association between m6A and responsiveness to antitumor therapy. m6A modification of DCP2 induced by METTL3 further promotes mitochondrial autophagy and leads to chemoresistance in small-cell lung cancer 15. YTHDF1 inhibits T cells through the m6A-p65-CXCL1 axis, leading to immunotherapy resistance in BLCA 16. However, studies systematically exploring the predictive role of m6A in the prognosis and treatment responsiveness of BLCA are relatively absent.

In this study, we identified two m6A subtypes in the TCGA-BLCA cohort by unsupervised clustering using 23 m6A-related genes (MRGs) as a starting point. The two m6A subtypes had significantly different biogenetic profiles and clinical characteristics. We quantified the chemotherapy sensitivity of patients by drug sensitivity analysis and explored the tumor immune infiltration landscape of BLCA by algorithms such as single sample gene set enrichment analysis (ssGSEA) and ESTIMATE. We used Kaplan-Meier (K-M) survival analysis, Cox regression analysis, and chi-square test to reveal significant differences in the prognosis and treatment responsiveness of patients with different m6A subtypes. Finally, we constructed a robust classifier by random forest (RF) and artificial neural network (ANN) that can accurately classify BLCA patients into different m6A subtypes. Our findings are expected to provide new strategies for individualized treatment for patients with BLCA.

## Materials and methods

### Data collection and processing

The TCGA-BLCA cohort data were downloaded from the GDC database (https://portal.gdc.cancer.gov/), including gene expression profiling data for 425 samples (19 normal bladder samples and 406 BLCA samples) and clinical information for 412 samples (Supplementary Table S1). For further analysis, the gene expression profile data were converted from fragments per kilobase million (FPKM) format to transcripts per kilobase million (TPM) format and filtered to obtain the protein-coding genes. The final expression matrix in log2 (TPM+1) format was obtained for a total of 19,492 genes. Simple nucleotide variation (SNV) data of the TCGA-BLCA cohort were downloaded from the GDC database in ".maf" format and used to calculate the tumor mutation burden (TMB) value for each sample based on the formula: TMB (mut/mb) = total mutation amount (including synonymous, non-synonymous, substitution, insertion, and deletion mutations)/size of target coding area. Gene copy number variation (CNV) data derived from 415 samples of the TCGA-BLCA cohort were retrieved from the UCSC Xena database (https://xenabrowser.net/datapages/). Data for the m6A subtypes validation cohort GSE87304 were obtained from the Gene Expression Omnibus (GEO) database (https://www.ncbi.nlm.nih.gov/geo/). 23 MRGs were derived from previous studies 17, 18

### Exploring the genetic and biological significance of MRGs in BLCA

The "limma" R package was used to perform a gene expression matrix-based difference analysis to examine mRNA expression differences between normal bladder samples and BLCA samples in the TCGA-BLCA cohort. The samples were categorized into low and high-expression groups based on the optimal cutoff values of the gene expression profiles, and the "survival" and "survminer" R packages were used to perform K-M survival analyses with the log-rank test to compare the differences in overall survival (OS) and progression-free survival (PFS) between the two groups. The frequencies of gain and loss of MRGs in BLCA were counted based on CNV data, and the corresponding positions on chromosomes were visualized by the "Rcircos" R package.

### Unsupervised clustering and evaluation based on MRGs

The "ConsensusClusterPlus" R package was used to perform unsupervised consensus clustering based on the expression profiles of 23 MRGs to classify TCGA-BLCA samples into m6A subtypes with different MRGs expression profiles. Re-sampling 80% of the samples 50 times using coalescent pam clustering with Euclidean distance. K-M survival analysis was used to compare differences in OS and PFS between the m6A subtypes. Box plots were used to compare the expression differences of 23 MRGs between the m6A subtypes. pCA and t-SNE were used for downscaling the expression profiles to visualize the distinguishability of the expression characteristics of MRGs between the subtypes. The "GSVA" R package was used to perform gene set variant analysis (GSVA) based on "c2.cp.kegg.v7. 5.1. symbols.gmt" (from the KEGG database), 70 pathways closely related to metabolism were screened as reference gene sets to compare the variation of metabolic pathways between the subtypes.

### Genetic variation analysis based on the m6A subtypes

The "maftools" R package was used to analyze SNV data in ".maf" format from the TCGA-BLCA cohort and to present the somatic mutation landscape of the m6A subtypes in the form of waterfall plots. RNA stemness scores (RNAss) data were obtained from the Pan-Cancer Atlas Hub (https://pancanatlas.xenahubs.net) 19 to characterize the tumor cell stemness levels of the samples. Expression levels of four mismatch repair genes (MSH2, MSH6, MLH1, and PMS2) were used to characterize the microsatellite instability (MSI) of the samples. Differences in TMB, RNAss, and the expression of four mismatch repair genes were compared between the m6A subtypes by difference analysis.

### Evaluation of cellular infiltration in TME

A marker gene set of 23 immune cells 20 was downloaded from the TISIDB database (http://cis.hku.hk/TISIDB/data/download/CellReports.txt). The relative infiltration abundance of immune cells in the TME of patients in the TCGA-BLCA cohort was assessed by the ssGSEA algorithm. The MCP counter-algorithm was used to assess the infiltration abundance of fibroblasts in TME. The "ESTIMATE" R package was used to calculate the StromalScore, ImmuneScore, and ESTIMATEScore of TME. Where StromalScore and ImmuneScore characterize the proportions of the stromal and immune components, respectively. ESTIMATEScore is the sum of the two and is negatively correlated with tumor purity 21. Lists of cytokines 22 and immune checkpoint genes 23 were collected from previous literature to compare the differences in cytokine and immune checkpoint gene expression between the m6A subtypes.

### Clinical subgroup analysis

TCGA typing of BLCA is an important molecular typing in which patients are classified into five subtypes: Basal squamous, Luminal, Luminal infiltrated, Luminal papillary, and Neuronal. The Luminal papillary has the best prognosis, and the Neuronal Neuronal has the poorest prognosis. The three Luminal types are insensitive to cisplatin while Basal squamous is sensitive to cisplatin. The luminal infiltrated type is sensitive to immunotherapy. The subtype distribution map demonstrated the differences in the distribution of the five TCGA subtypes between the m6A subtypes. "Stage" and "grade" are recognized as important clinical subgroup characteristics that are closely related to the prognosis of BLCA. We counted and compared the distribution of patients with various grading and staging characteristics in different m6A subtypes and visualized them in pie charts. Univariate and multivariate Cox regression analyses were used to explore the effects of m6A subtype, grade, and stage on patients' OS and PFS, as well as the clinical independence of the effects.

### Treatment responsiveness analysis

Chemotherapy, radiotherapy, and immunotherapy are important treatment options for patients with BLCA. The "pRRophetic" R package predicted the 50% inhibitory concentration (IC50) values of several common chemotherapeutic agents for BLCA based on the "cgp2016" dataset 24. The "oncoPredict" R package predicted the IC50 and area under curve (AUC) values of several common chemotherapeutic agents for BLCA based on the "GDSC2" dataset 25. The smaller the predicted IC50 and AUC values, the more sensitive the patient is likely to be to the drug. The radiotherapy-related efficacy assessment statistics based on the TCGA-BLCA cohort compared the response to radiotherapy in patients with different m6A subtypes. The Tumor Immune Dysfunction and Exclusion (TIDE) database (http://tide.dfci.harvard.edu/) was used to predict the response of the patients to immunotherapy. Higher scores of TIDE, Exclusion, Dysfunction, Cancer associated fibroblasts (CAF), MDSC, and lower MSI scores represented poorer response to immunotherapy.

### Construction of ANN classifier

First, we analyzed the differential gene expression profiles of different m6A subtypes and obtained subtype differential genes (SDEGs). | logFC |> 1 and p <0.05 as the screening threshold, the p-value is corrected for FDR. Subsequently, we used the "randomForest" R package to perform the RF algorithm to further screen for signature genes. The default number of iterations for the RF is 100. The RF model was considered robust enough when the 500 trees were constructed. Genes were scored for importance based on the Gini coefficient and genes with an importance score greater than 2 were screened as classifier genes. Further, we compared the classifier gene expression levels of a single sample to the median across all samples. In subtype B up-regulated genes, the value was 1 if the expression level was higher than the median and 0 otherwise. The opposite was true in subtype B down-regulated genes. The patients' gene expression profiles were transformed into [0,1] standardized "gene signature". Finally, we constructed an ANN classifier using the "neuralnet" R package based on the "gene signature" and visualized it with the "NeuralNetTools" R package 26. The number of hidden layer neurons was set to two-thirds of the number of input layer neurons plus two-thirds of the number of output layer neurons. Receiver operating characteristic (ROC) curves were constructed to predict the predictive accuracy of the ANN classifier using the "pROC" R package. The GSE87304 cohort was used to validate the clinical significance of the ANN classifier.

### Screening of key model genes

Difference analysis was used to examine mRNA expression differences of 13 model genes between normal bladder samples and BLCA samples in the TCGA-BLCA cohort. Single-gene K-M survival analyses with the log-rank test were performed to compare the differences in OS and PFS between the high-expression group and the low-expression group. Immunohistochemistry based on The Human Protein Atlas database (HPA, https://www.proteinatlas.org) for comparing protein expression levels of key model genes between normal bladder tissue and BLCA tissue.

### Statistical analysis

All bioinformatics analyses in this study were performed using R (version 4.2.1), and the Perl language was used for batch processing and cleaning of data. Unless otherwise stated, the difference analysis in this study was performed using the "limma" R package, with the Wilcoxon test used to compare differences between two groups and the Kruskal-Wallis test used to compare differences among three or more groups. The chi-square test was used to compare differences in rates and component ratios between groups. K-M survival analysis and log-rank test were used to compare differences in survival rates between groups. A two-tailed p-value of <0.05 was considered statistically significant.

## Results

### Identification and evaluation of BLCA subtypes based on 23 MRGs

**Figure 1:**
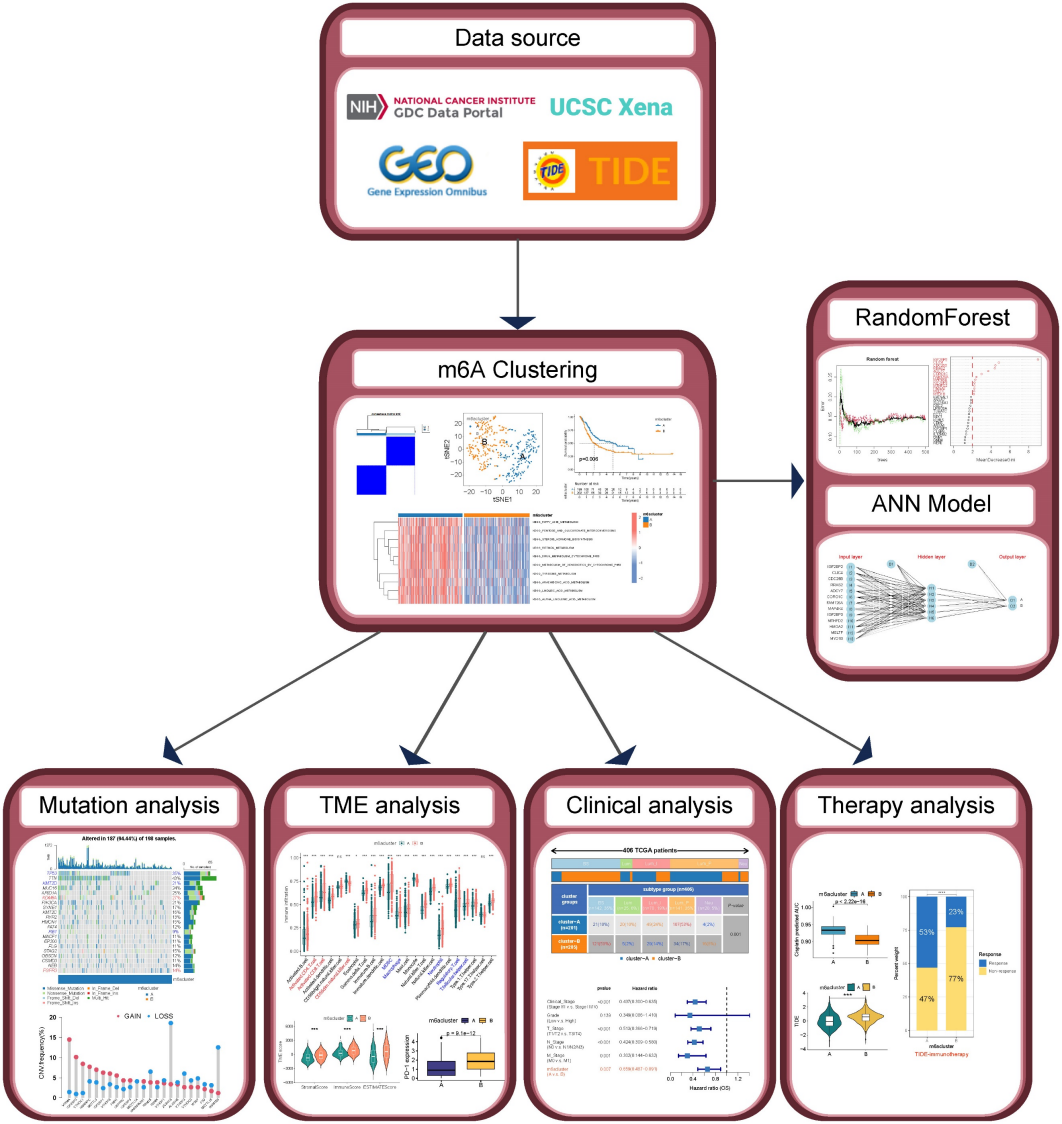
The workflow of this study.

**Figure 2:**
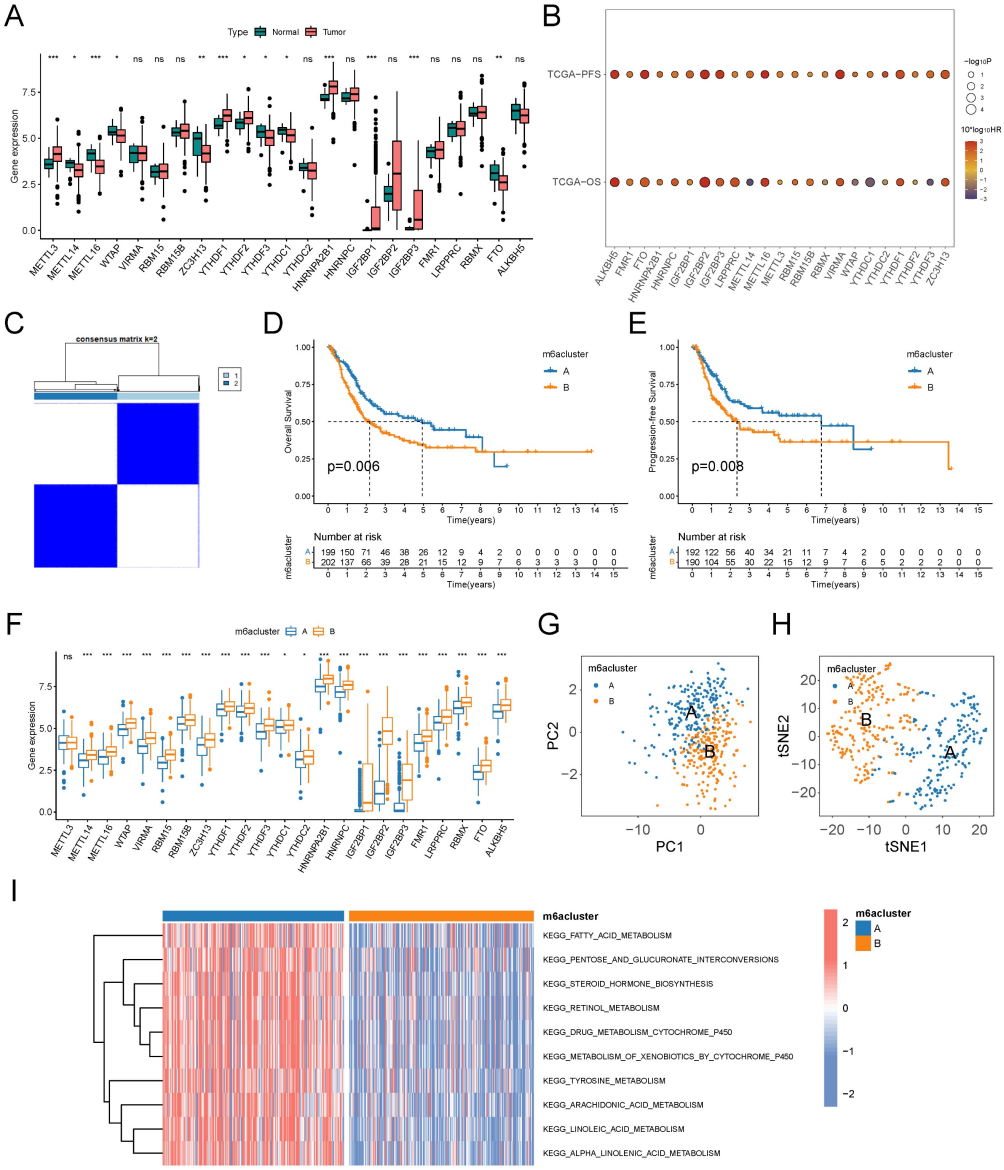
Identification and evaluation of m6A Subtypes based on 23 MRGs in TCGA-BLCA cohort. (A) Difference analysis of mRNA expression level of 23 MRGs between BLCA and normal bladder tissue. (B) Log-rank test of single-gene K-M survival analysis based on optimal cutoff value grouping. The larger the sphere, the smaller the p-value. Red represents prognostic risk factors and purple represents prognostic protective factors. (C) Unsupervised consensus clustering divides BLCA samples into two clusters (k=2) based on 23 MRGs. (D-E) OS curves (D) and PFS curves (E) for the two subtypes of patients with BLCA. (F) Expression differences analysis of 23 MRGs between the two subtypes. (G-H) PCA (G) and tSNE (H) show a significant difference in transcriptomes of MRGs between the two subtypes. (I) GSVA between the two subtypes with the metabolic pathway gene sets in KEGG. The color of the bar represents the GSVA score. ns, no significant difference; *p<0.05; **p<0.01; ***p<0.001.

The workflow of this study is shown in Figure 1. We performed a difference analysis of the expression profiles of 23 MRGs between BLCA tissue and normal bladder tissue based on the TCGA-BLCA cohort, which showed that the mRNA expression levels of METTL3, YTHDF1, YTHDF2, HNRNPA2B1, IGF2BP1, and IGF2BP3 were significantly higher in BLCA tissue than in normal bladder tissue, whereas the mRNA expression levels of METTL14, METTL16, WTAP, ZC3H13, YTHDF3, YTHDC1, and FTO in contrast (Figure 2A). The results of the log-rank test for single-gene K-M survival analysis showed that all 23 MRGs were risk factors for PFS in patients with BLCA, while for OS, several were prognostic-protective factors. Genes such as ALKBH5, FTO, IGF2BP2, IGF2BP3, and IGF2BP3 were significant risk factors for OS and PFS (Figure 2B). Unsupervised clustering based on the expression profiles of 23 MRGs classified BLCA patients into two m6A subtypes, A and B (Figure 2C). The prognosis of patients with the two m6A subtypes was significantly different, and the OS (Figure 2D) and PFS (Figure 2E) of subtype B were significantly lower than those of subtype A. The expression levels of almost all MRGs in subtype B were significantly higher than those of subtype A (Figure 2F, except for METTL3), and the results of PCA (Figure 2G) and tSNE (Figure 2H) verified that there were a significant distinguishable MRGs expression profiles between the two subtypes. GSVA results based on the KEGG metabolic gene set demonstrated the 10 metabolic pathways that differed most between the two subtypes, with significant activation of fatty acids, arachidonic acid, linoleic acid, and linolenic acid pathways in the A subtype compared with the B subtype (Figure 2I).

### Genetic variation analysis based on m6A subtypes

We next analyzed the genetic variation associated with m6A subtypes from multiple perspectives. The waterfall plots showed that the two m6A subtypes had significantly different somatic mutation landscapes, with subtype A having significantly lower mutation frequencies of the TP53, KMT2D, and RB1 and significantly higher mutation frequencies of the KDM6A, and FGFR3 compared with subtype B (Supplementary Figure S1A, S1B). There was no significant difference in TMB between the two m6A subtypes (Supplementary Figure S1C). Using RNAss to characterize tumor cell stemness, we found no significant difference in tumor cell stemness between the two subtypes similarly (Supplementary Figure S1D). However, the expression levels of all four mismatch repair genes were significantly lower in subtype A than in subtype B (Supplementary Figure S1E). All 23 MRGs had varying degrees of CNV gain and loss frequencies, with VIRMA having the highest gain frequency and RBM15B having the highest loss frequency (Supplementary Figure S1F, S1G).

### Exploration of TME based on m6A subtypes

**Figure 3:**
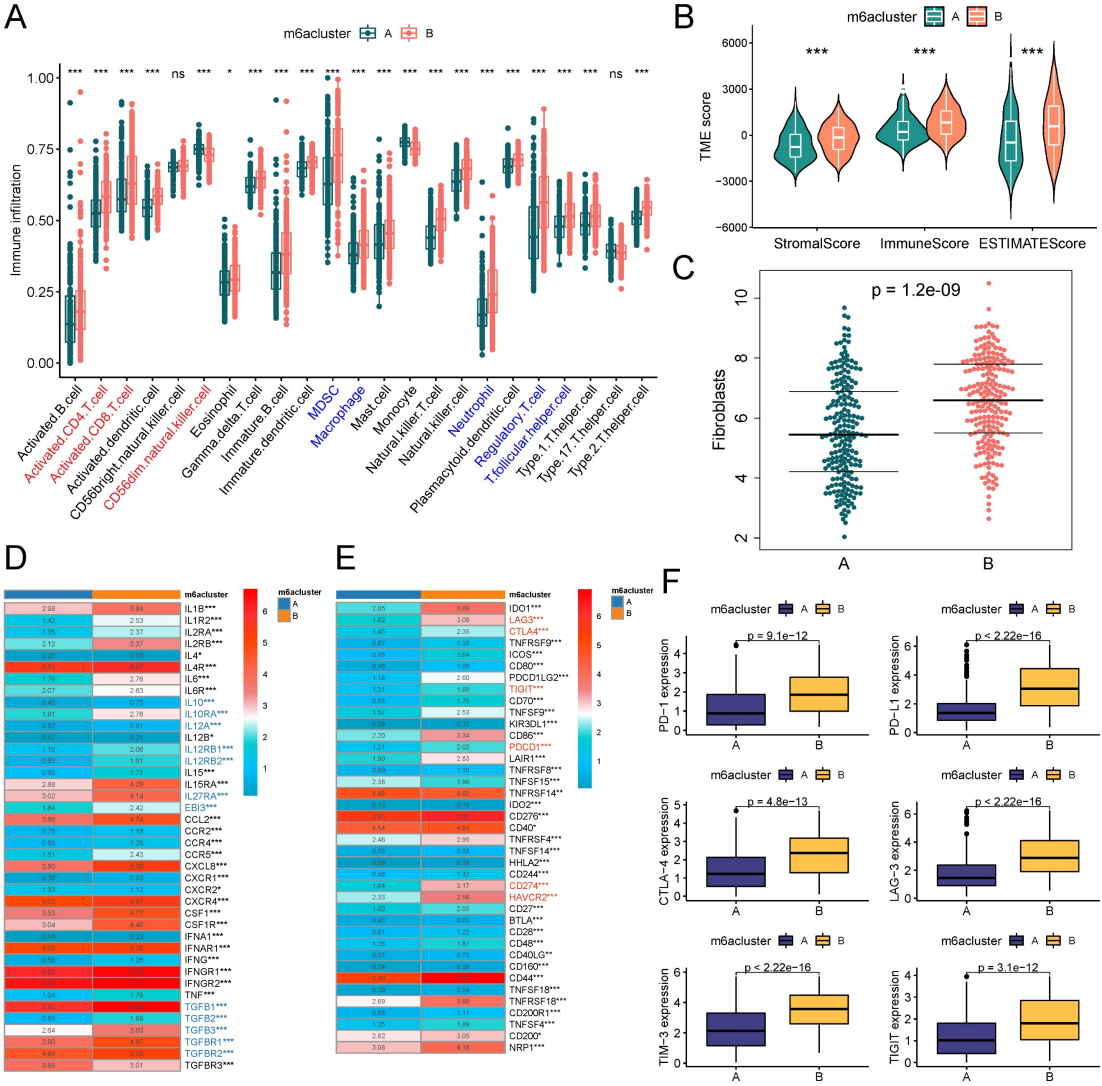
m6A subtypes related TME analysis based on TCGA-BLCA cohort. (A) Box plots on the ssGSEA algorithm illustrated immune infiltration landscapes in the TME of patients in two m6A subtypes. Blue represents immunosuppressive cells and red represents immune effector cells. (B) Violin plot for difference comparison of StromalScore, ImmuneScore, and ESTIMATEScore between the two m6A subtypes. (C) Prediction of fibroblast infiltration abundance in the TME of two subtypes based on MCPcounter algorithm. (D) Expression differences of cytokine genes between the two m6A subtypes. Blue represents classical inhibitory cytokines. (E-F) Expression differences of immune checkpoint genes between the two m6A subtypes (E), six common immune checkpoint genes have lower levels in subtype A (F). ns, no significant difference; *p<0.05; **p<0.01; ***p<0.001.

A growing body of evidence demonstrates that TME is an important factor influencing the prognosis and treatment responsiveness of patients with tumors. First, we evaluated the infiltration abundance of 23 immune cells in TME using the ssGSEA algorithm. The results showed that immunosuppressive cells, such as MDSC and Treg cells, all had significantly up-regulated infiltration abundance in the B subtype. Among immune effector cells, CD56dimNK cell infiltration abundance was significantly down-regulated in subtype B, while activated CD4T cells and activated CD8T cells were significantly up-regulated in subtype B (Figure 3A). We comprehensively assessed the stromal and immune components in the TME of patients by the ESTIMATE algorithm, and the StromalScore, ImmuneScore, and ESTIMATEScore were significantly higher in subtype B than in subtype A (Figure 3B). Fibroblasts were closely associated with both stromal deposition and immunosuppression in TME, and the results of the MCPcounter algorithm suggested that the level of fibroblast infiltration in TME was significantly higher in subtype B than in subtype A (Figure 3C). Further, we explored the expression of cytokines in the TME of patients with both m6A subtypes and found that the expression level of immunosuppressive cytokines was significantly higher in patients with subtype B than subtype A (Figure 3D). The expression landscape of immune checkpoints suggested that most of the immune checkpoint molecules were expressed at significantly higher levels in the TME of subtype B (Figure 3E), including the most common ones in the clinic, namely PD-1, PD-L1, CTLA-4, LAG-3, TIM-3, and TIGIT (Figure 3F).

### Characterization of clinical features associated with m6A subtypes

**Figure 4:**
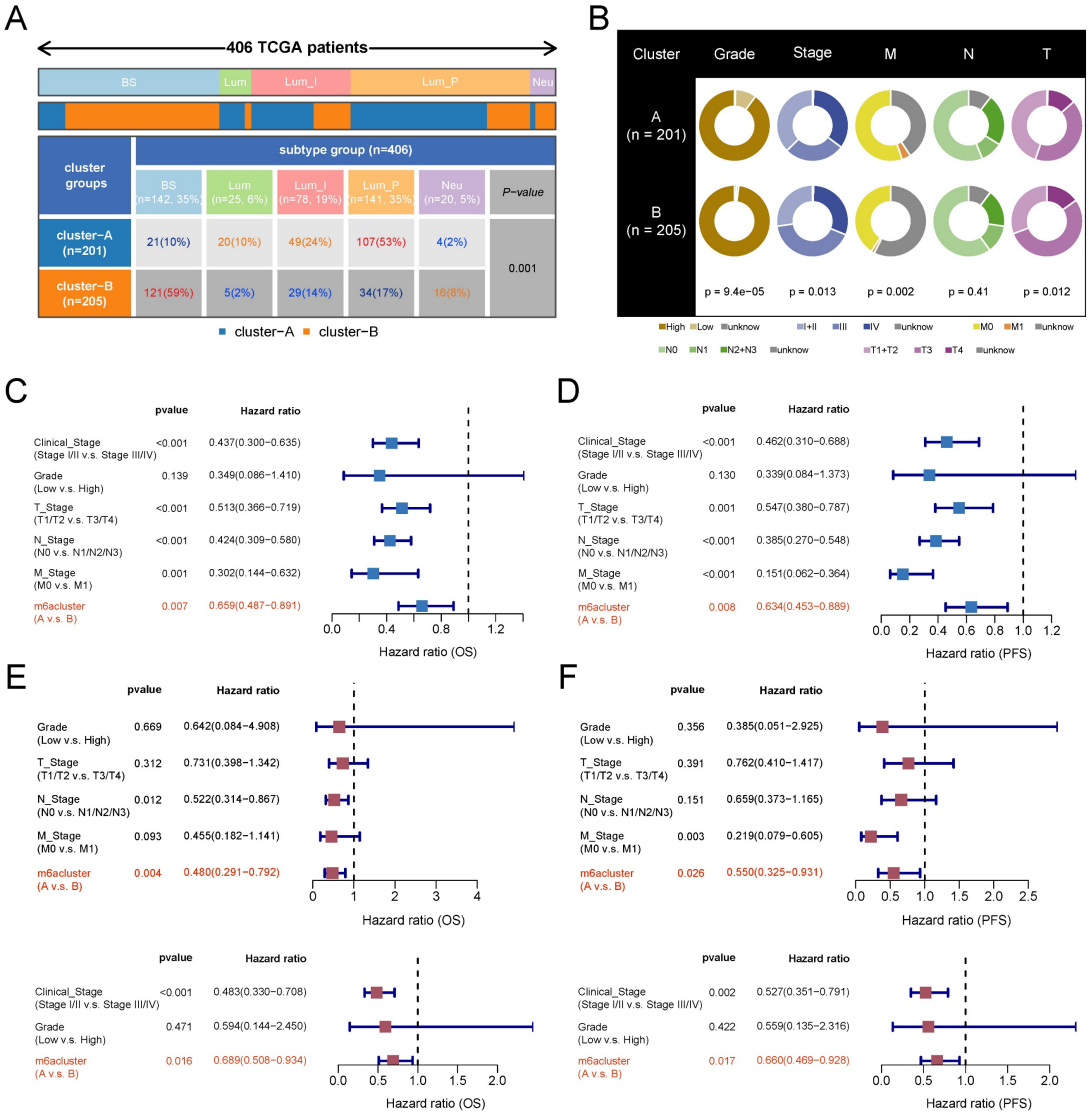
m6A subtypes related clinical characteristics analysis based on TCGA-BLCA cohort. (A) Distribution proportion of five molecular subtypes in two m6A subtypes. Red and orange represent high proportions, blue represents low proportions. (B) Pie charts show differences in the distribution of various clinical features in the two m6A subtypes, and chi-square tests are performed. (C-D) Univariate Cox regression analysis of m6A subtypes combined with other clinical features based on OS (C)and PFS (D). (E-F) Multivariate Cox regression analysis of m6A subtypes combined with other clinical features based on OS (E)and PFS (F). BS, Basal squamous; Lum, Luminal; Lum_I: Luminal infiltrated; Lum_P, Luminal papillary; Neu, Neuronal.

To explore the clinical significance of m6A subtypes in patients with BLCA, we analyzed them in combination with TCGA subtypes. The luminal papillary subtype had the highest proportion of patients with subtype A and was significantly higher than subtype B. The Basal squamous subtype had the highest proportion of patients with subtype B and was significantly higher than subtype A (Figure 4A). Grading and staging are important clinical features of tumors. We found that low-grade patients were mainly clustered in subtype A. The proportion of patients with clinical stage I and II was higher and the proportion of stage III was lower in subtype A than in subtype B. The proportion of patients with stage T1 and T2 was higher and the proportion of stage T3 was lower in subtype A than in subtype B. The distributions of N and M stages were not significantly different between the two subtypes (Figure 4B, with a lot of missing data for the M stage). We next verified the clinical independence of m6A subtypes in predicting prognosis by Cox regression analysis. After excluding the interference of the remaining grading and staging factors, the m6A subtype still independently predicted patients' prognostic risk (Figure 4C-4F).

### m6A subtypes predict responsiveness to antitumor therapy

**Figure 5:**
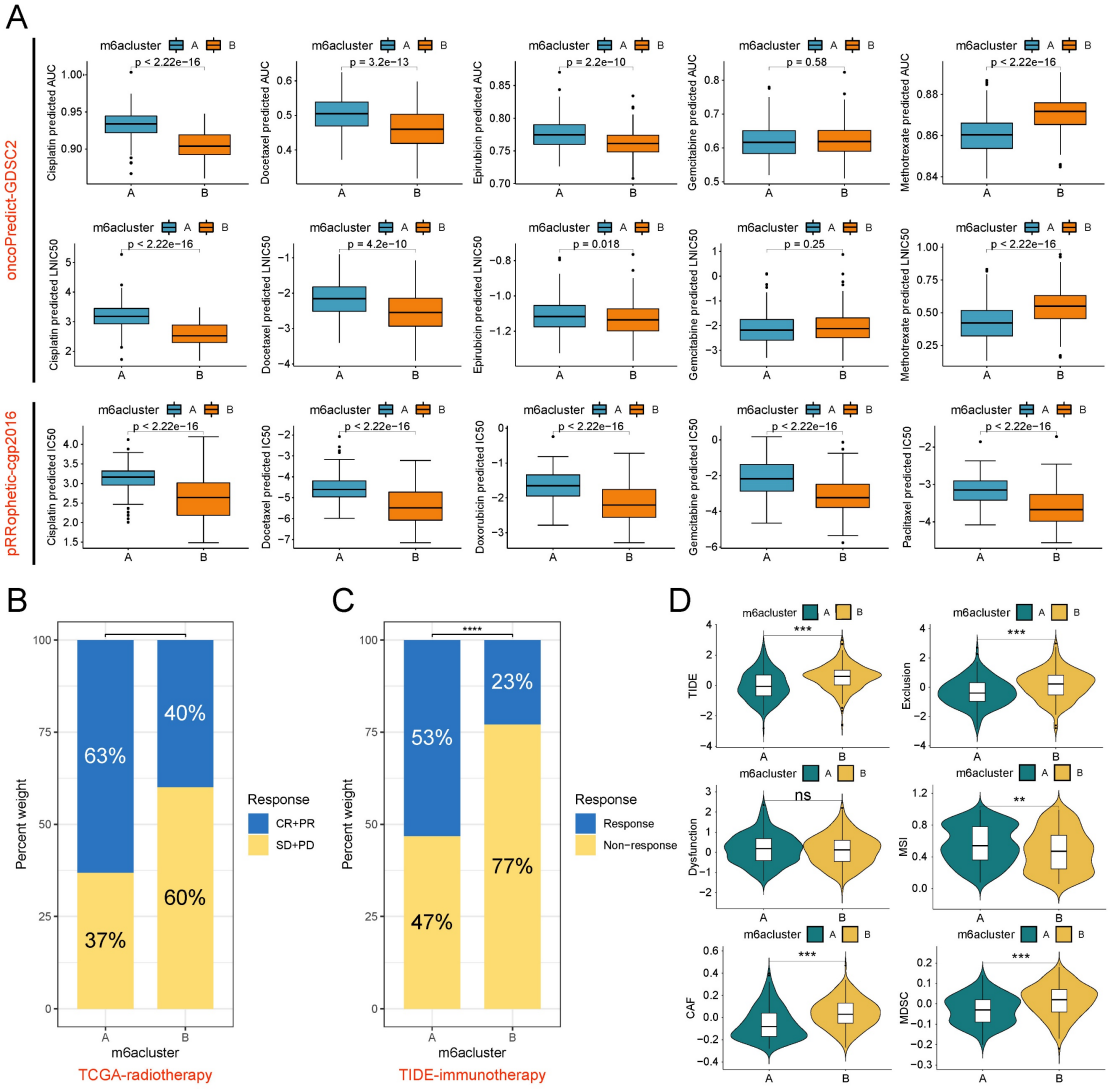
Therapy responsiveness analysis based on m6A subtypes. (A) The oncoPredict algorithm (based on the GDSC2 data source) and pRRophetic algorithm (based on the cgp2016 data source) was used to predict the IC50 and AUC value of two m6A subtypes to chemotherapy drugs in the TCGA-BLCA cohort. (B) Proportional distribution of radiotherapy response status in the two m6A subtypes based on TCGA-BLCA-radiotherapy cohort. (C) Proportional distribution of immunotherapy response status in the two m6A subtypes based on TCGA-BLCA-TIDE cohort. (D) Difference analysis of 6 kinds of scores closely associated with immune escape between the two m6A subtypes based on the TIDE algorithm. ns, no significant difference; **p<0.01; ***p<0.001; ****p<0.0001. CR, Complete response; PR, Partial response; SD, Stable disease; PD, Progressive disease.

Radiotherapy, chemotherapy, and immunotherapy are important options for patients with BLCA. The results of drug sensitivity analysis showed that subtype B exhibited lower IC50 values and AUC values for cisplatin and docetaxel symbolizing higher sensitivities in both pRRophetic and oncoPredict algorithms than subtype A. The single-algorithm prediction showed higher sensitivities for epirubicin, doxorubicin, and paclitaxel and lower methotrexate sensitivities for subtype B compared with subtype A. However, gemcitabine had contradictory results in two algorithms (Figure 5A). In the radiotherapy cohort of TCGA, patients with subtype B had lower proportions of complete response (CR) and partial response (PR) and higher proportions of stable disease (SD) and progression disease (PD) compared with subtype A (Figure 5B). Based on the TIDE algorithm to assess immunotherapy responsiveness in the TCGA-BLCA cohort, patients with subtype A had significantly higher immunotherapy response rates than patients with subtype B (Figure 5C). Meanwhile, patients with subtype A had lower TIDE scores, Exclusion scores, CAF infiltration scores, and MDSC infiltration scores, and higher MSI scores than patients with subtype B (Figure 5D).

### Construction and validation of ANN-based m6A classifier

**Figure 6:**
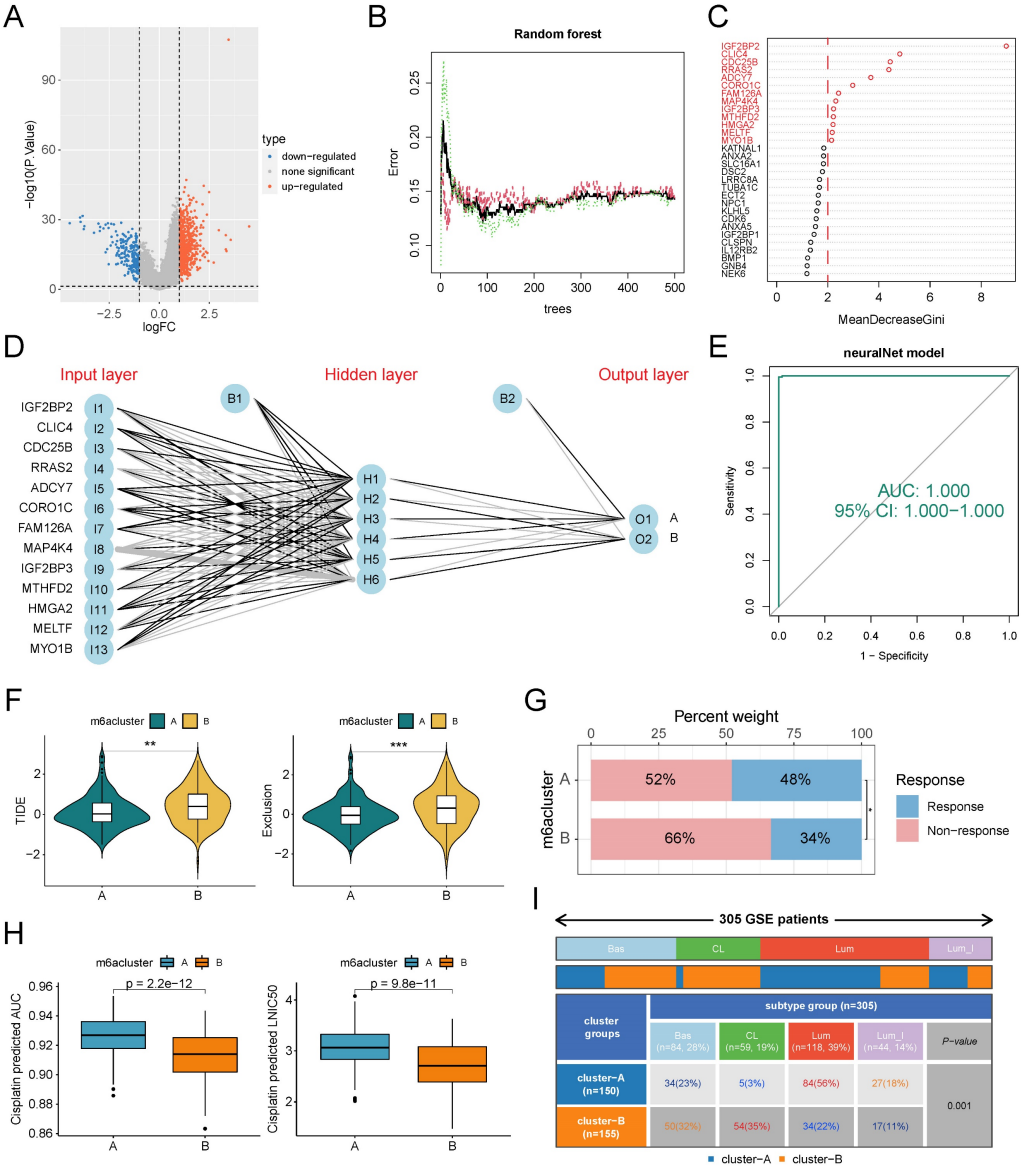
Construction and validation of ANN classifiers for identification of m6A subtypes. (A) Difference analysis identified SDGs of subtype B compared with subtype A. (B) The relationship between the number of trees and model error in random forest. The model has the smallest error when the number of trees is 264. Green for subtype B, red for subtype A, black for all samples. (C) The genes whose importance score was greater than 2 based on the Gini coefficient method were selected as classifier genes. (D) Schematic diagram of ANN classifier, where the number of hidden layers is 6. (E) The ROC of the ANN classifier was used to verify the predictive efficacy. (F-I) Patients in the GSE87304 cohort were classified into two m6A subtypes by the ANN classifier. (F) Difference analysis of TIDE score and Exclusion score between the two m6A subtypes. (G) Proportional distribution of immunotherapy response status in the two m6A subtypes based on GSE87304-TIDE cohort. (H) Difference analysis of the IC50 and AUC value to cisplatin between the two subtypes based on the oncoPredict algorithm. (I) Distribution proportion of four molecular subtypes in two m6A subtypes. *p<0.05; **p<0.01; ***p<0.001. Bas, Basal; CL, Claudin Low; Lum, Luminal; Lum_I: Luminal infiltrated.

One drawback of unsupervised clustering is that the clustering results will be unstable when the number of samples is small. To map the clustering results from large samples to small samples or even single samples for clinical applications, we mined the genetic features of m6A subtypes and developed a classifier. First, we obtained 890 SDEGs by difference analysis. Using subtype A as a control, subtype B has 207 down-regulated genes and 683 up-regulated genes (Figure 6A). Subsequently, we used the random forest algorithm to screen the SDEGs for features (Figure 6B), and the model error was minimized when 90 trees were constructed. The genes with gene importance score greater than 2 were selected as subtype feature genes, which were IGF2BP2, CLIC4, CDC25B, RRAS2, ADCY7, CORO1C, FAM126A, MAP4K4, IGF2BP3, MTHFD2, HMGA2, MELTF, and MYO1B (Figure 6C). The ANN classifier was constructed using these 13 genes as the input layer and the two m6A subtypes as the output layer (Figure 6D). Weight parameters between nodes in ANN model were showed in Supplementary Table S2. ROC analysis showed that this classifier recognized the two m6A subtypes with an AUC value of 1 (Figure 6E). The GSE87304 cohort was categorized into two m6A subtypes using this classifier. In this cohort, patients with subtype A had lower TIDE scores and Exclusion scores (Figure 6F) and higher immunotherapy response rates (Figure 6G). Patients with subtype B had lower drug sensitivity-related IC50 values and AUC values for cisplatin than patients with subtype A (Figure 6H). This is consistent with previous results. Finally, we found that patients with subtype B had a higher proportion of basal types, especially claudin low types (a subclass of basal types), and a lower proportion of luminal types than patients with subtype A (Figure 6I).

### Screening of key model genes

**Figure 7:**
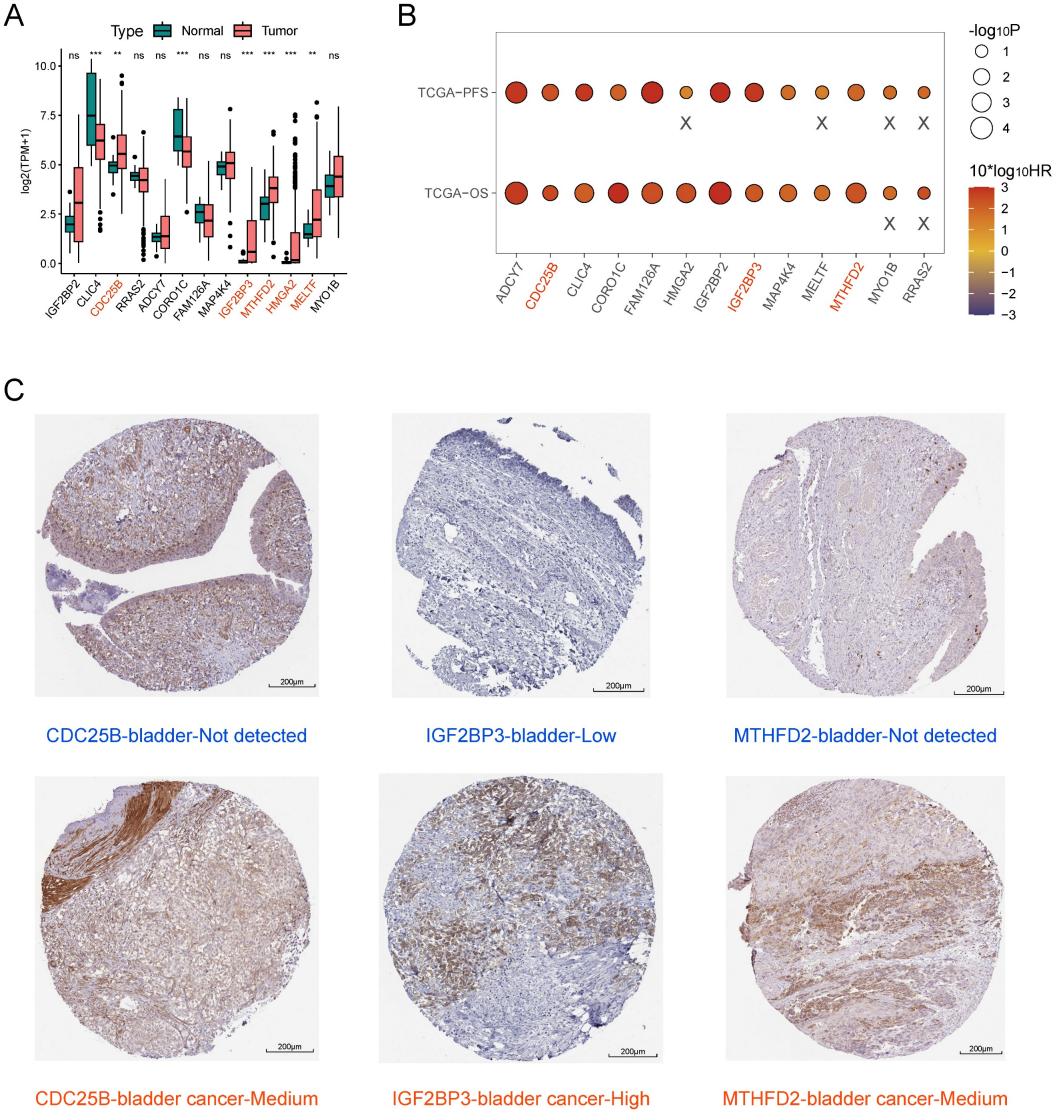
Screening of key model genes. (A) Difference analysis of mRNA expression level of 13 model genes between BLCA and normal bladder tissue. Those marked in red are five potential oncogenes. (B) Log-rank test of single-gene K-M survival analysis based on optimal cutoff value grouping. "X" represents p > 0.05. Those marked in red are three potential oncogenes that are risk factors for both OS and PFS regarded as key model genes. (C) Comparison of protein expression levels of three key model genes between normal bladder tissue and BLCA tissue by immunohistochemistry based on the HPA database.

We combined the results of difference analysis (Figure 7A) and single-gene K-M survival analyses (Figure 7B) to screen key model genes. We found all 13 model genes were prognostic risk factors for BLCA. CDC25B, IGF2BP3, MTHFD2, HMGA2, and MELTF were significantly higher expressed in BLCA tissue than in normal bladder tissue. On this basis, patients with high expression of CDC25B, IGF2BP3, and MTHFD2 had significantly lower OS and PFS compared with the low-expression group. Immunohistochemistry results showed that the protein expression levels of these three model genes were higher in BLCA tissue than in normal bladder tissue (Figure 7C).

## Discussion

BLCA is a morphologically and genomically heterogeneous disease with a wide range of histological subtypes and associated molecular alterations^27^. While traditional surgical treatment, radiotherapy, and platinum-containing chemotherapy remain important cornerstones in the management of patients with BLCA, emerging therapies, including targeted therapies and immunotherapies, represent a paradigm shift in the management of treatment for BLCA^28^, ^29^. Various anti-PD-1/PD-L1 agents have demonstrated significant benefits in combination with platinum-containing chemotherapy, and new-generation targeted therapies associated with FGFR3 alterations, tumor cell expression of nectin-4 and trophoblast surface antigen 2 (TROP2) have likewise been rapidly incorporated into clinical practice 4, 30, 31. However, issues of prognosis and treatment responsiveness heterogeneity continue to plague clinicians. TCGA project identified luminal and basal molecular subtypes and genetic drivers of muscle invasive BLCA with different treatment responses 27, but they also have limitations.

m6A modification is the methylation that occurs at the N6 position of adenosine and is the most prevalent internal modification on eukaryotic mRNAs 5. An increasing number of studies have focused on the association of m6A with tumor metabolism 6, 32, tumor microenvironment, immune escape^33^, and the significance of m6A modifications in targeted cancer therapy^34^, ^35^. We found that 23 classical MRGs had significant differences in expression, prognosis, and genetic variation in BLCA. It may be a good idea to analyze them systematically.

We classified patients with TCGA-BLCA into two m6A subtypes based on the expression profiles of 23 MRGs: A and B. The expression levels of almost all MRGs in subtype B were significantly higher than those in subtype A, suggesting that subtype B tumors may have higher levels of m6A modification. Based on the results of survival analysis, drug sensitivity analysis, and TIDE analysis, we broadly outlined the characteristics of clinical prognosis and responsiveness to antitumor therapy of the two m6A subtypes. First, patients with subtype B had a significantly worse prognosis (lower OS and PFS) compared with subtype A, and the m6A subtype was a prognostic predictor of BLCA independent of staging and grading. Second, patients with subtype B may have lower responsiveness to immunotherapy and responsiveness to radiotherapy but higher sensitivity to cisplatin and docetaxel compared with subtype A. Third, by comparing the somatic mutation landscapes of the two m6A subtypes, we found that patients with subtype A have a significantly higher rate of FGFR3 mutations and may have a higher rate of benefit to the pan-FGFR inhibitor erdafitinib.

Comparison with TCGA subtyping partially explains the reasons for this difference. Overall, the proportion of the three luminal types was higher in patients with subtype A, with the Luminal papillary subtype being the highest. In contrast, the proportion of the basal squamous subtype was highest in patients with subtype B. Patients with the luminal type had a better prognosis and were more sensitive to immunotherapy but less sensitive to chemotherapy, especially cisplatin, compared with the basal squamous subtype. This is consistent with the clinical characteristics of the m6A subtype.

Tumour m6A modifications are strongly associated with immunosuppressive TME 36, 37. This may be one of the reasons for the poor response to immunotherapy in B subtype patients with high m6A modification levels. We carved out the TME landscapes of patients with both subtypes and found that TME in patients with subtype B showed high CAF-like immunosuppressive features: high levels of stromal deposition, immunosuppressive cell infiltration, and expression of inhibitory cytokines and immune checkpoint factors. In addition, patients with subtype B had higher mismatch repair gene expression and lower MSI scores. While TMB and RNAss were not significantly different between the two subtypes. This suggests that MSI may be a factor in the difference in responsiveness to immunotherapy, whereas TMB and tumor stemness are not.

Further, we mined the characteristic genes of m6A subtypes and developed a classifier based on RF and ANN, and the results of ROC analysis showed that the classifier could accurately classify BLCA patients into two m6A subtypes. Results based on the GEO dataset further validated the predictive ability of m6A subtypes for cisplatin sensitivity and immunotherapy responsiveness. Previous bioinformatics research has focused on the field of risk modeling, where risk scores are calculated to predict the prognosis and responsiveness to antitumor therapy of patients. However, the biggest drawback of this approach is the inability to determine the threshold for dividing the population because of the heterogeneity of different detections. In this study, we propose for the first time to construct a molecular subtype classifier by the random forest combined with an artificial neural network, which can classify patients into different m6A subtypes very accurately. Our findings may identify BLCA patients with different prognostic and treatment responsiveness profiles, providing more precise individualized treatment strategies for them.

There are some limitations of this study. On the one hand, we need more prognostic and therapeutic data from real-world cohorts to validate the predictive ability and clinical value of the m6A subtype classifier. On the other hand, it remains to be elucidated which molecular mechanisms mediate the differences in clinical characteristics between the m6A subtypes, and the results of GSVA showed that the activation of metabolic pathways differed between the two subtypes. There may be complex crosstalk between m6A modification, tumor immunity, and tumor metabolism.

## Conclusion

In this study, we developed an ANN classifier for identifying BLCA patients with different m6A subtypes, and patients with different m6A subtypes have significantly different prognosis and responsiveness to antitumor therapy. The findings of this study provide novel personalized treatment strategies for patients with BLCA.

## Supplementary Material

Supplementary figure and tables.

## Abbreviations

ANN: Artificial neural network
AUC: Area under curve
BLCA: Bladder cancer
CAF: Cancer associated fibroblasts
CNV: Copy number variation
CR: Complete response
FPKM: Fragments per kilobase million
GEO: Gene Expression Omnibus
GSVA: Gene set variant analysis
HPA: Human protein atlas
IC50: 50% inhibitory concentration
K-M: Kaplan-Meier
m6A: N6-methyladenosine methylation
MRGs: m6A-related genes
MSI: Microsatellite instability
OS: Overall survival
PFS: Progression-free survival
PD: Progression disease
PR: Partial response
RF: Random forest
RNAss: RNA stemness scores
ROC: Receiver operating characteristic
SD: Stable disease
SDEGs: Subtype differential genes
SNV: Simple nucleotide variation
ssGSEA: single sample gene set enrichment analysis
TCGA: The Cancer Genome Atlas
TIDE: Tumor Immune Dysfunction and Exclusion
TMB: Tumor mutation burden
TME: Tumor microenvironment
TPM: Transcripts per kilobase million
