# Supplementary material for: Development of an m6A subtype classifier to guide precision therapy for patients with bladder cancer
Source: J Cancer. 2024 Aug 13;15(16):5204–17. doi: 10.7150/jca.99483 (PMC11375535; doi:10.7150/jca.99483)
Supplement: Supplementary file 1 — Supplementary figure and tables. [file jcav15p5204s1.pdf]

Supplementary Legends

Supplementary FigS1 Exploration of the potential correlation between m6A subtypes and genetic variation. (A-B) The waterfall chart shows the landscape of gene somatic cell mutations in patients with subtype A (A)and subtype B (B). Red represents a significant percentage increase, blue represents a significant percentage decrease. (C) Differences in TMB between patients with two subtypes. (D) Differences in RNAss that symbolize cellular stemness between patients with two subtypes. (E) Differences in the expression level of four mismatch repair genes between patients with two subtypes. (F) Frequencies of gain and loss of CNV in 23 MRGs. (G) The Chromosome localization and the landscape of CNV in 23 MRGs. ***p<0.001.

Supplementary TabS1 Baseline Data Sheet for the Clinical Cohort of TCGA-BLCA.

Supplementary TabS2 Weight parameters between nodes in ANN model.

Supplementary FigS1

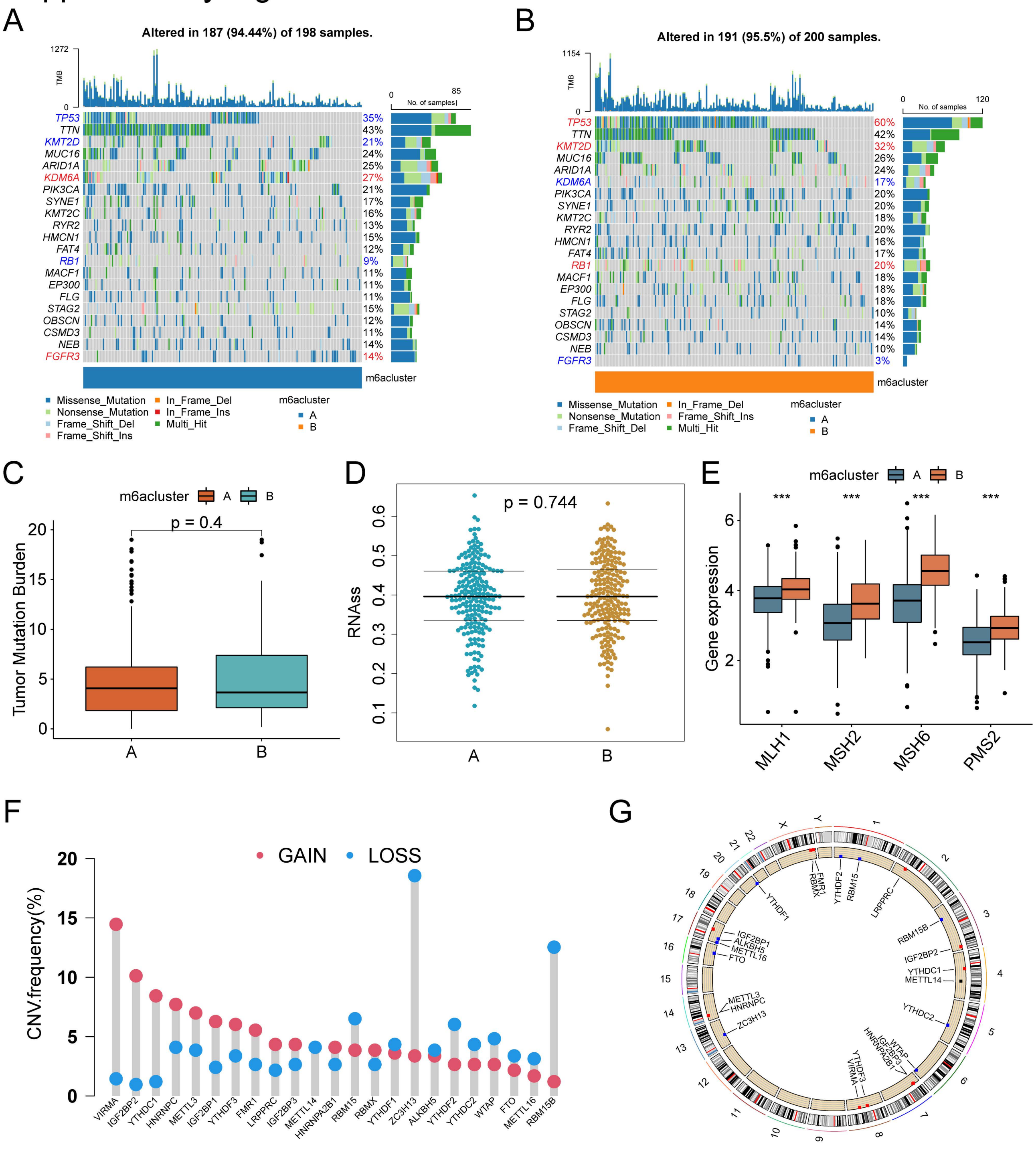

Table S1 | Baseline Data Sheet for the Clinical Cohort of TCGA-BLCA.

Characteristic	Levels	Numbers (%)
Age	≤65 years old	162 (39.3%)
	>65 years old	250 (60.7%)
Gender	Male	304 (73.8%)
	Female	108 (26.2%)
Grade	Low	21 (5.1%)
	High	388 (94.9%)
T_Stage	T1	3 (0.7%)
	T2	152 (37.0%)
	T3	197 (47.9%)
	T4	59 (14.4%)
N_Stage	N0	239 (64.6%)
	N1	47 (12.7%)
	N2	76 (20.5%)
	N3	8 (2.2%)
M_Stage	M0	196 (94.7%)
	M1	11 (5.3%)
Clinical_Stage	I	2 (0.5%)
	II	131 (31.9%)
	III	141 (34.4%)
	IV	136 (33.2%)

Table S2 | Weight parameters between nodes in ANN model.

		Hidden layer						
		Intercept	HL1	HL2	HL3	HL4	HL5	HL6
Input layer	Intercept	-	2.19E+00	3.93E+01	-3.32E+00	2.91E+01	2.29E+00	-2.98E+00
	IGF2BP2	-	3.90E+00	-3.33E+01	-1.07E+01	-1.83E+01	8.17E+01	-2.36E+01
	CLIC4	-	4.66E-01	9.62E-01	9.53E-02	-2.99E+00	-6.48E+00	1.36E+01
	CDC25B	-	7.18E+00	-8.25E-02	-3.14E+00	-1.80E-01	2.21E+01	-3.04E+01
	RRAS2	-	-2.86E+01	-3.04E+00	-6.96E+00	7.47E+00	-3.47E+01	-3.05E+01
	ADCY7	-	1.99E+01	-5.85E+00	3.46E+00	-1.66E+01	2.84E+01	2.79E+01
	CORO1C	-	-6.96E+00	9.55E-01	3.25E+00	2.36E+00	1.20E+00	-7.21E-01
	FAM126A	-	2.95E+01	2.01E+01	-2.79E-01	-8.56E+00	-5.49E+00	1.26E+01
	MAP4K4	-	-6.54E+01	-2.71E+01	-7.17E+00	1.81E+01	-3.65E+01	-7.64E+02
	IGF2BP3	-	-7.25E+00	-7.14E+00	3.30E+00	-2.16E+01	1.58E+01	-4.41E+00
	MTHFD2	-	3.94E+00	-4.87E+00	-5.61E-01	-1.21E+01	4.21E+00	-1.07E+01
	HMGA2	-	9.22E+00	-6.04E+00	3.38E+00	-1.08E+01	1.53E+01	1.45E+01
	MELTF	-	-6.03E+00	9.49E+00	-2.51E-01	8.73E+00	-1.03E+01	4.85E-01
	MYO1B	-	6.27E+00	1.74E+00	4.30E-01	-1.43E+01	-1.36E+01	-3.30E+00
Output layer	A	1.02E+00	1.01E+00	-1.01E+00	2.08E+00	9.88E-01	-1.02E+00	-1.10E+00
	B	-1.86E-02	-1.01E+00	1.01E+00	-2.08E+00	-9.88E-01	1.02E+00	1.10E+00
